# Supplementary material for: Sex Differences in Durability Following Heavy Intensity Cycling Exercise in Trained Athletes
Source: Scand J Med Sci Sports. 2026 Apr 9;36(4):e70273. doi: 10.1111/sms.70273 (PMC13063221; doi:10.1111/sms.70273)
Supplement: Supplementary file 1 — Table S1: Descriptive statistics for the absolute values of cardiopulmonary and neuromuscular data measured in an unfatigued and fatigued state. [file SMS-36-e70273-s001.docx]

Table S1. Descriptive statistics for the absolute values of cardiopulmonary and neuromuscular data measured in an unfatigued and fatigued state.

|  | |  | Males | |  | Females | |  |
| --- | --- | --- | --- | --- | --- | --- | --- | --- |
|  | |  | Unfatigued | Fatigued | Difference | Unfatigued | Fatigued | Difference |
| GET | | PO (W) | 226 ± 42 | 191 ± 52 | -35±33 | 135 ± 20 | 131 ± 17 | -5±16 |
|  | | V̇O_2_ (ml·kg^-1^·min^-1^) | 38.7 ± 5.6 | 36.2 ± 6.8 | -2.5±5.2 | 32.2 ± 3.7 | 32.4 ± 2.9 | -1.9±7.5 |
|  | | HR (bpm·min^-1^) | 147 ± 12 | 143 ± 11 | -4±9 | 143 ±11 | 146 ±7 | 3±9 |
| RCP | | PO (W) | 331 ± 51 | 290 ± 59 | -41±32 | 222 ± 35 | 208 ± 33 | -13±19 |
|  | | V̇O_2_ (ml·kg^-1^·min^-1^) | 52.2 ± 6.6 | 48.6 ± 6.8 | -3.6±3.9 | 45.5 ± 4.6 | 43.7 ± 4.1 | -4.7±12 |
|  | | HR (bpm·min^-1^) | 174 ± 12 | 169 ± 13 | -5±6 | 172± 14 | 170 ± 10 | -2±9 |
| V̇O_2peak_ | | V̇O_2_ (ml·kg^-1^·min^-1^) | 58.3 ± 6.8 | 53.5 ± 7.7 | -4.8±4.9 | 51.2 ± 3.1 | 48.5 ± 4.7 | -2.6±2.8 |
|  | | V̇O_2_ (L·min^-1^) | 4.40 ± 0.60 | 4.03 ± 0.61 | -0.37±0.41 | 2.91 ± 0.35 | 2.74 ± 0.39 | -0.15±0.17 |
|  | | PO (W) | 410 ± 56 | 358 ± 64 | -53±37 | 270 ± 35 | 249 ± 39 | -20±21 |
|  | | Bla (mmol^-1^) | 10.2 ± 3.1 | 4.9 ± 1.2 | -5.0±2.8 | 9.9 ± 1.7 | 5.6 ± 2.2 | -4.3±3 |
|  | | HR_max_(bpm·min^-1^) | 185 ± 17 | 184 ± 10 | -1±9 | 184 ± 10 | 183 ± 10 | -1±10 |
|  | | RER | 1.22 ± 0.06 | 1.08 ± 0.08 | -0.14±0.09 | 1.19 ± 0.05 | 1.11 ± 0.08 | -0.1±0.1 |
|  | | V̇E (L·min^-1^) | 184 ± 18 | 151 ± 32 | -33±30 | 118 ± 9 | 108 ± 14 | -11±13 |
| NMF | | MVIC (N) | 630 ± 148 | 504 ± 107 | -126±73 | 373 ± 75 | 323 ± 76 | -50±58 |
|  | | Db_100_ (N) | 248 ± 81 | 203 ± 73 | -45±35 | 170 ± 51 | 141 ± 38 | -21±22 |
|  | | Db_10_ (N) | 247 ± 76 | 152 ± 59 | -96±50 | 164 ± 44 | 112 ± 33 | -41±31 |
|  | | Q_tw·pot_ (N) | 168 ± 58 | 121 ± 46 | -47±29 | 108 ± 29 | 76 ± 23 | -26±14 |
|  | | VA (%) | 94 ± 4 | 88 ± 7 | -6±6 | 91 ± 5 | 85 ± 9 | -6±6 |
|  | | M_max_ (mV) | 10.6 ± 5.8 | 9.8 ± 7.0 | -0.78±2.2 | 7.0 ± 4.5 | 8.1 ± 4.7 | 0.9±1.5 |
|  |  |  |  |  |  |  |  |  |
